# Disruption of the HLA-E/NKG2X axis is associated with uncontrolled HIV infections

**DOI:** 10.3389/fimmu.2022.1027855

**Published:** 2022-11-18

**Authors:** Luis Romero-Martín, Clara Duran-Castells, Mireia Olivella, Míriam Rosás-Umbert, Marta Ruiz-Riol, Jorge Sanchez, Dennis Hartigan-O´Connor, Beatriz Mothe, Àlex Olvera, Christian Brander

**Affiliations:** ^1^ IrsiCaixa AIDS Research Institute, Hospital Germans Trias i Pujol, Institute for Health Science Research Germans Trias i Pujol (IGTP), Badalona, Spain; ^2^ Universitat Autonoma de Barcelona (UAB), Barcelona, Spain; ^3^ University of Vic-Central University of Catalonia (UVic-UCC), Vic, Spain; ^4^ CIBERINFEC, Centro de Investigación Biomédica en Red, Instituto de Salud Carlos III, Madrid, Spain; ^5^ Impacta, Lima, Peru; ^6^ California National Primate Research Center and Department of Medical Microbiology and Immunology, University of California, Davis, Davis, CA, United States; ^7^ Division of Experimental Medicine, Department of Medicine, University of California, San Francisco, San Francisco, CA, United States; ^8^ Fundació Lluita contra la Sida, Infectious Disease Department, Hospital Universitari Germans Trias i Pujol, Badalona, Spain; ^9^ ICREA, Barcelona, Spain

**Keywords:** HIV, Natural Killer cells, HLA-E, NKG2A, NKG2C

## Abstract

The contribution of the HLA-E/NKG2X axis in NK-mediated control of HIV infection remains unclear. We have studied the relationship between HLA-E expression and phenotypical as well as functional characteristics of NK cells, in the context of chronic HIV infection and in an *in vitro* model of acute infection. High viremia in HIV+ individuals was related to increased HLA-E expression, and changes in NK subpopulations, especially a reduction of the CD56^bright^ as well as an increase in adaptive NK subpopulation. Uncontrolled HIV infection was also characterized by a reversion of the NKG2A/NKG2C expression ratio and a loss of positive and negative regulation of NK mediated by HLA-E. This was reflected in a lower cytotoxic, degranulation and cytokine production capacity, especially in CD56^bright^ and adaptive NK. In line with these results, HLA-E expression showed a positive correlation with viral growth inhibition in an *in vitro* model of acute infection at day 7, which was lost after 14 days of culture. Using HLA-E expressing K562 cells, we determined that only one out of 11 described HIV-derived HLA-E epitopes increased HLA-E surface stability. In spite of that, eight of the 11 epitopes were capable of increasing degranulation and three drove differences in NK-cell mediated cell lysis or cytokine secretion. In conclusion, our results indicate that HLA-E molecules presenting HIV-derived epitopes may sensitize target cells for NK lysis in early HIV infection. However, prolonged exposure to elevated HLA-E expression levels *in vivo* may lead to NK cell dysfunction and reduced viral control In chronic infection.

## Introduction

Natural killer (NK) cells are innate lymphoid cells that represent approximately 5-10% of circulating peripheral blood mononuclear cells (PBMC) and can be divided in a broad spectrum of NK-cell subsets. In brief, NK cells were first classified into two subpopulations based on the expression of CD56 and CD16, named CD56^bright^ (CD56^bright^ CD16^-^) and CD56^dim^ (CD56^dim^ CD16^+^), and different phenotypical and functional characteristics were attributed to these subsets (1). More recent studies have reported a novel, memory-like, adaptive NK subset that expresses the maturation marker CD57 and responds specifically to certain pathogens (2). In addition, in persistent infections, sustained NK stimulation can lead to a skewed exhausted phenotype with reduced cytokine production (3–5). To date, NK cell exhaustion has been mainly studied in the context of tumor-associated NK cells and to application of strategies to reverse their exhaustion and augment anti-tumor activity (6–9).

In general, NK-cell stimulation is the result of a balance between activating and inhibitory signals to which they are exposed, signals that can vary depending on the nature of the binding cell (10). NK cells express diverse receptors, which usually use classical or non-classical MHC-class I molecules as ligands. They can be divided in inhibitory receptors, which trigger NK activation if they cannot detect MHC molecules presenting canonical self-derived peptides on the surface of target cells (referred to as “the missing self”), and their activating counterparts that activate NK cells upon recognition of MHC class I molecules presenting non-self-peptides (11). The C-type lectin-like receptor superfamily (NKG2) are NK receptors with most of its members (NKG2A, B, C, E and H) binding non-classical MHC class Ib molecules (including HLA-E). Binding of HLA-E to the heterodimer formed by CD94 and NKG2A has an inhibitory effect on NK cell function, since NKG2A contains two immune receptor tyrosine-based inhibitory motifs (ITIM) that transduce an inhibitory signal (12). However, if HLA-E is downregulated on target cells (the missing self), or the bound epitope repertoire on HLA-E prevents effective interaction with CD94/NKG2A, the loss of ligand interaction induces NK activation due to a lack of the inhibitory signal (7, 8). On the other hand, when CD94 forms heterodimers with NKG2C, E or H, these receptors have an activating effect on the NK cell. This different function is due to a positively charged residue within the transmembrane regions of NKG2C, E and H that allows them to associate with the immunoreceptor tyrosine-based activating motif (ITAM) of DAP12 (13). Of note, NKG2C expression is increased at later stages of NK cell maturation and binds HLA-E with lower affinity than NKG2A (14). Among the myriad of NK receptors, we focused our study on these two NK receptors (NKG2A and NKG2C) due to their marked opposite, but very relevant function in regulating NK function through the HLA-E/NKG2X axis.

HLA-E is a non-classical class Ib histocompatibility molecule with very limited polymorphism. Only two alleles (HLA-E*01:01 and HLA-E*01:03) have been demonstrated to be properly expressed on the cell surface and together cover >99% of the global allelic diversity of HLA-E (15). Given this conservation, targeting HLA-E epitope presentation has been considered for developing universal immunotherapies and vaccines (16). Structural differences between the dominant HLA-E alleles are very limited since a single residue in position 107 within the α2 domain, differentiates HLA-E*01:01 (arginine) from HLA-E*01:03 (glycine) (17). The presence of the positive amino acid in this position in HLA-E*01:01 is thought to cause the lower surface stability of HLA-E*01:01 compared to HLA-E*01:03 (18). In addition, this change may also induce a subtle structural shift in the binding-cleft α2 helix, resulting in an altered repertoire of presented epitopes. HLA-E was first described as part of the innate immune system and was reported to inhibit NK mediated lysis of healthy cells by presenting peptides derived from the leader sequences of HLA class I molecules to the CD94/NKG2A heterodimer (19). Growing evidence supports now also a role of HLA-E in the adaptive immune response through the presentation of pathogen-derived epitopes to specific CD8+ T-cells (20, 21).

Several viruses have developed mechanisms to evade NK killing of infected target cells, such as the expression of peptides mimicking human canonical self-epitopes to effectively stabilize inhibitory NK receptors (22) or the modulation of surface molecules, such as HLA class I, to counteract NK recognition *via* the missing self-axis (23). For instance, HCMV-derived UL40 peptide, presented on HLA-E, transduces an inhibitory signal through CD94/NKG2A recognition (24). HCMV has also been described to alter NK cells subsets by driving the proliferation of a memory-like CD57^+^NKG2C^high^ NK cells subset, with an imprint in the *IFNG* locus that leads to a strong IFN-γ production upon NKG2C engagement (25, 26). Finally, it has been shown that the NKG2A/NKG2C ratio is inverted in NK cells derived from people living with HIV and co-infected with HCMV (27).

A number of past studies support an important role of HLA-E and NK cells activity also in the context of HIV infection. First, the HIV Nef protein has been suggested to downregulate surface expression of HLA-E, impairing the recognition of infected cells by either the T cell receptors or NKG2X/CD94 and thereby avoiding cell lysis (28). Still, mostly through elution studies, five HIV-1 derived epitopes have been reported to be presented by HLA-E: RMYSPVSIL (RL9), PEIVIYDYM (PM9), TALSEGATP (TP9), RIRTWKSLV (RV9) (29) and AISPRTLNA (AA9) (30), while some others have been reported as potential HLA-E epitope candidates since they show slight effects on HLA-E stabilization: EKIKALVEI (EI9), MYSPVSILD (MD9), NEEAAEWDR (NR9), QMAVFIHNF (QF9) and YFSVPLDEG (YG9) (29). However, among these HIV-1-derived HLA-E binding peptides, only AA9 has been shown to have a functional impact on the NK response by increasing the activation of NKG2A^+^ NK cells. These data have been interpreted to suggest that HIV-1 might alter the presented HLA-E peptide repertoire and trigger NK cells degranulation by blocking the inhibitory signal of the NKG2A receptor (30). Thus, understanding the role of HLA-E/NKG2X regulation in the NK mediated elimination of HIV-1 infected cells and how the availability and presentation of viral epitopes can interfere in these processes, may be critical to our understanding of HIV control. These mechanisms may be especially relevant when considering proposed NK cell therapies, such as the infusion of *ex vivo* expanded specific NK cell populations and chimeric antigen receptor (CAR)-expressing NK cells (31, 32), or the use of latency reversing agents to eliminate HIV infected cells by Kick-and-Kill strategies (33).

In the present work, we hypothesized that an increased HLA-E surface expression in untreated, chronically HIV-1 infected, viremic individuals leads to a chronic NK stimulation through the HLA-E/NKG2X axis, while HIV-derived epitopes could further modulate the HLA-E/NKG2X interaction. Such modulation may eventually cause a disruption of the balance between NKG2A and NKG2C expression and induce an impaired NK response. We employed peptide binding studies, NK functional tests and structural analysis of HLA-E*01:01 and HLA-E*01:03 to test this hypothesis and to address how the HLA-E/NKG2X axis relates to NK activity and consequent clinical outcome of untreated HIV infection.

## Material and methods

### Cell samples

HIV-infected subjects (n=51) recruited at the Hospital Germans Trias i Pujol (Badalona, Spain) and the IMPACTA clinics in Lima (Peru) were included in this study. These included individuals that were not receiving antiretroviral treatment and had i) low HIV plasma viral loads (HIV-low, n=31) of <10,000 HIV RNA copies/mL (range: 25 to 9,999; median: 1,877 HIV RNA copies/mL) and CD4 counts that ranged from 434 to 1,343 cells/mm^3^ (median: 762 cells/mm^3^) or ii) high HIV plasma viral loads (HIV-high, n=20) of >50,000 HIV RNA copies/mL (range: 50,295 to 1,200,000; median: 239,511 HIV RNA copies/mL) and CD4 counts that ranged from 11 to 726 cells/mm^3^ (median: 284 cells/ mm^3^) (**Supplementary Table 1**). Additional unrelated cohorts were included as comparison and validation groups (n=54): i) highly-exposed seronegative individuals, sampled six months prior to infection (n=6, pre-HIV), ii) acute/recently HIV-1 infected individuals, sampled within a median of 6 months from HIV-1 acquisition (n=8, acute), iii) low risk HIV seronegative individuals (n=24, seronegative), iv) chronically HIV infected individuals one year before receiving cART (n=11, chronics), v) chronically infected individuals after receiving cART for at least one year (n=6, treated) and an additional validation cohort of long-term non-progressors, HIV infected individuals not receiving cART (n=23, LTNP) (**Supplementary Table 2**). The study was approved by the Comitè Ètic d’Investigació Clínica of Hospital Germans Trias i Pujol (CEIC EO-12-042), and all participants provided written informed consent. Fresh leukocyte fraction from blood donations (n=18) from HIV-seronegative individuals was obtained from the Catalan Blood and Tissue Bank (Banc de Sang i Teixit, BST).

### HIV-derived HLA-E binders peptides selection

A total of 14 HLA-E binding epitopes (**Table 1**) were synthesized (Snypeptide) and tested in our study, including: 2 described canonical HLA-E epitopes, one derived from the HLA-Cw1 signal peptide (VL9-Cw1) (41) and another derived from CMV (VL9-CMV) (42), and 12 non-canonical peptides derived from EBV (SL9) (43) and HIV (AA9, KG9, MD9, PM9, QE9, RL9, RV9, SN9, TP9, VI9, YG9). HIV-derived peptides were selected based on reported HLA-E surface stabilization activity (MD9, PM9, RV9, TP9, YG9) (29), alteration of NK degranulation (AA9) (30) and HIV homologies of described SIV epitopes shown to be the targeted by SIV-specific Mamu-E restricted T-cell responses in rhesus macaques (KG9, QE9, RL9, SN9, VI9) (44).

**Table 1:** HLA-E binding epitopes tested in this study.

ID	Sequence	Organism	Protein			Reference
VL9-B7	VMAPRTVLL	Human	HLA-B7		Leader sequence	34
VL9-Cw1	VMAPRTLIL	Human	HLA-Cw1		Leader sequence	35
VL9-CMV	VMAPRTLIL	CMV		UL40		36
E SL9	SQAPLPCVL	EBV		BZLF1		37
HIV AA9	AISPRTLNA	HIV	Gag		p24	38
HIV KG9	KRIKCFNCG	HIV	Gag		p24	39
HIV MD9	MYSPVSILD	HIV	Gag		p24	40
HIV PM9	PEIVIYQYM	HIV	Pol		Retrotranscriptase	40
HIV QE9	QMLKETINE	HIV	Gag		p24	39
HIV RL9	RMYSPTSIL	HIV	Gag		p24	40
HIV RV9	RIRTWKSLV	HIV		Vif		40
HIV SL9	SEELRSLYN	HIV	Gag		p24	39
HIV TP9	TALSEGATP	HIV	Gag		p17	40
HIV VI9	VGEIYKRWI	HIV	Gag		p24	39
HIV YG9	YFSVPLDEG	HIV	Pol		Protease	40

### Structural analysis of HLA-E and TCR/NKG2 receptors interaction

A molecular model was constructed for HLA-E*01:01 and HLA-E*01:03 bound to PM9 and RL9 peptides based on the 6GL1 X-ray crystal structure. The initial molecular model of HLA-E-peptides interacting with NKG2A/2C-CD94 and TCR was constructed based on available X-ray crystal structure in the Protein Data Bank (3CDG and 5W1V). The initial models were Energy Minimized using Gromacs 5.0 (45).

### Peptide-pulse HLA-E surface stabilization assay

HLA-E*(01:03) transfected K562 cell lines (HLA-E/VL9-K562), co-expressing the HLA-B*0702 signal sequence derived VL9 peptide (VMAPRTVLL) (46), were kindly provided by Dr. Joosten (University of Leiden, The Netherlands) and originally produced by Dr. E. Weiss (Ludwig-Maximilians-Universitat, Munich, Germany) (42). These cell lines were cultured in IMDM supplemented with 10% fetal bovine serum (FBS) and 200 µg/mL of G418 (I10 medium) at 26°C 5% CO_2_ for three hours, prior to being pulsed with 20 µg/mL of canonical and non-canonical HIV-derived peptides, for 16 hours at 26°C 5% CO_2_. Cells were then washed twice with I10 medium and rested for 2 hours at 37°C 5% CO_2_. Cells were then stained with 3D12 PE anti-human HLA-E (BioLegend) and fixed (Fix&Perm Cell Permeabilization Kit, Thermofisher). At least 100,000 events were collected on a LSRII BD cytometer and analyzed using FlowJo v10 software. HLA-E surface stabilization was expressed as (% HLA-E+ cells in peptide-pulsed cells/% HLA-E+ cells in non-pulsed cells) and (HLA-E MFI in peptide-pulsed cells/HLA-E MFI in non-pulsed cells).

### NK cytotoxicity assay

Peripheral blood mononuclear cells (PBMC) were isolated from cryopreserved leukocyte fractions using Lymphoprep™ (Stemcell) density gradient. NK cells were isolated by magnetic bead separation (MACS Miltenyi Biotec) from PBMC, following the manufacturer’s instructions. NK cytotoxic capacity was measured at 10:1, 5:1, 2.5:1, and 1:1 NK effector to target cell ratio (HLA-E*0103/VL9-K562 targets). Target cells were pulsed with canonical and non-canonical HIV-derived peptides and labeled using calcein-AM to perform a retention assay as previously described (47). Briefly, 12,500 peptide-pulsed HLA-E/VL9-K562 cells were labelled for 30 minutes at 37°C 5% CO_2_ with calcein-AM at 10 nM and then cultured in R10 medium (RPMI supplemented with 10% FBS, 100 U/mL penicillin and 100 ug/mL streptavidin), together with the different ratios of NK cells isolated from the HIV seronegative individuals, for 4 hours at 37°C and 5% CO2. Cultures were then run on a Canto II BD Cytometer and the percentage of killing of labelled target cells calculated as 100-% calcein-AM^+^ K562 cells. In each condition, an exponential regression curve was determined by the percentage of killing resulting from the different effector to target ratios in the co-culture. Results were expressed in lytic units (48) calculated as the number of NK cells contained in 10^6^ PBMC able to lyse 20% of 100,000 target cells.

### NK degranulation assay

NK degranulation activity was measured by flow cytometry at a fix 5:1 NK effector to target cell ratio. Targets were HLA-E*01:03/VL9-K562 cells pulsed with canonical and non-canonical (HIV-derived) peptides. NK cells sorted from cryopreserved PBMC and peptide-pulsed target cells were co-cultured for 4 hours in the presence of Golgi Stop, Golgi Plug and APC anti-human CD107 (Biolegend) and stained for surface markers: SK7 Amcyan anti-CD3 (BD), 5.1H11 BV785 anti-CD56 and B73.1 BV-605 anti-CD16 (Biolegend). Subsequently, cells were fixed, permeabilized (Fix&Perm Cell Permeabilization Kit, Thermofisher) and stained with 6401.1111 FITC anti-human TNF-α and B27 PE-Cy7 anti-human IFN-γ (BD). Approximately 20,000 events per samples were acquired on a Canto II BD Cytometer and analyzed using FlowJo v10 software.

### NK cells subpopulations and determination of NKG2A and NKG2C expression

To discriminate different NK cell subpopulations, isolated NK cells from cryopreserved PBMC from HIV seronegative (n=12), HIV-high (n=8) and HIV-low (n=8) individuals were stained with SK7 Amcyan anti-CD3 (BD), 5.1H11 BV-785 anti-CD56, B73.1 BV-605 anti-CD16, HNK-1 PerCP-Cy5 anti-CD57 (Biolegend), 131411 PE anti-NKG2A and 134591 APC anti-NKG2C (R&D Systems). Samples were run on a LSRII BD Cytometer and analyzed using FlowJo v10 software. NK cell subpopulations were classified as (i) CD56^-^CD16^-^ (CD3^-^CD56^-^CD16^-^CD57^-^), (ii) CD56^dim^ (CD3^-^CD56^dim^CD16^+^CD57^-^), (iii) adaptive NK cells (CD3^-^CD56^dim^CD16^+^CD57^+^) and (iv) CD56^bright^ (CD3^-^CD56^bright^CD16^-^CD57^-^). NKG2A and NKG2C surface expression were evaluated in all NK subpopulations, by the gating strategy summarized in **Figure S1A**. This data set, together with data from CD3-negative cells from the sorted NK cell fraction used in the NK-VIA experiments, were downsampled to obtain 3,000 events/fcs file. An unsupervised tSNE dimensional reduction was performed on FlowJo 10.5.2 using the default settings of a nearest neighbours of 15 and a minimum Euclidean distance of 0.5. Number of clusters was determined by X-Shift (Number nearest neighbors: 132, Euclidean distance metric) and FlowSOM and was applied to determine the level of expression of CD56, CD16, CD57, NKG2A and NKG2C in a total of 11 clusters in the CD3-negative population. The abundance of each cluster was evaluated comparing samples from HIV seronegative, HIV-high and HIV-low individuals.

### NK-mediated viral inhibition assay

NK cell mediated viral inhibition activity was measured in 12 HIV-negative individuals. Briefly, the CD4+ T cell enriched fraction was obtained from freshly isolated PBMC using NK and CD8+ T-cell specific magnetic beads depletion (MACS NK Isolation kit and CD8+ T-cell isolation kit, Miltenyi Biotec) and stimulated with PHA (5 µg/mL) in R10. HLA-E surface expression was measured on the CD3+ CD4+ population by flow cytometry (as mentioned before) after three days of stimulation. Gating strategy is shown in **Figure S1B**. This fraction was then infected by spinoculation (720g for 2h at 25°C) with HIV-1 BaL and HIV-1 NL4-3 laboratory-adapted strains at a multiplicity of infection (MOI) of 0.01. On day 2, the day before starting the co-culture, one vial of cryopreserved PBMC from the same individual was thawed and cultured overnight with 20 U/mL of IL-2, prior to positive NK cells sorting by magnetic bead separation (MACS NK isolation kit, Miltenyi Biotec). The assay was performed in triplicates by culturing 50,000 HIV-infected or uninfected autologous CD4+ T-cells alone or in the presence of unstimulated autologous NK cells (2:1, 1:1, 1:2 and 1:10 NK effector to CD4 + T-cells target cell ratio). NK cells were previously rested for 2 hours at 37°C 5% CO_2_ after magnetic sorting in R10 medium, supplemented with 50 U/mL of IL-2 and 1 ng/mL of IL-15. After one week (day 7 of co-culture), half of the medium was collected and replaced with 50 µL of R10 supplemented with 100 U/mL of IL-2 and 2 ng/mL of IL-15. At day 14, the culture supernatant was harvested and NK cells from the long-term culture characterized by flow cytometry (as described before). HIV-1 p24 Gag concentration in the supernatant was measured at day 7 and day 14 by commercially available HIV-1 p24 ELISA kit (Innogenetics) in accordance with manufacturer’s instructions. Samples were read on a Perkin Elmer Ensight Plate reader. NK-mediated inhibition of viral replication was calculated as %inhibition = [(HIV-1 p24 Gag concentration in CD4+ T-cells cultured alone)-(HIV-1 p24 Gag concentration in CD4+ T-cells cultured with NK cells)]/(HIV-1 p24 Gag concentration in CD4+ T-cells cultured alone)x100.

### Total HIV-1 DNA quantification

Total HIV-1 DNA was quantified in PBMC lysates by droplet digital PCR (ddPCR) in duplicate as described previously (39). Briefly, two different primer/probe sets, annealing to the 5′ long terminal repeat and Gag regions, respectively were used to circumvent sequence mismatch in the individual´s proviruses, and the RPP30 housekeeping gene was quantified in parallel to normalize sample input. Raw ddPCR data were analyzed using the QX100 droplet reader and QuantaSoft v.1.6 software (Bio-Rad).

### DNA/RNA extraction and real-time PCR

DNA and RNA were simultaneously extracted using the AllPrep DNA/RNA Mini kit (QIAGEN) from cell pellets from the 51 HIV infected individuals and the 12 HIV seronegative individuals. Retrotranscription (SuperScript II first-strand synthesis Supermix) was performed on those samples and the resulting cDNA was used for reverse transcription-PCR using TaqMan gene expression assay for detection of HLA-E (Hs03045171_m1) and TBP (Hs99999910_m1) all from Applied Biosystems in a 7500 Real-Time PCR Systems (Applied Biosystems™). Relative expression was calculated as 2^-ΔCt^ (where Ct is the median threshold cycle from 3 replicates).

### HLA-E genotyping

An in-house developed strategy based on Next Generation Sequencing (NGS) was used for HLA-E genotyping of human PBMC and HLA-E expressing K562 cell lines at the Laboratori d’Histocompatibilitat i Immunogenètica, Banc de Sang i Teixits (BST). This strategy consists of a long-range PCR to amplify the HLA-E gene from 5’ UTR to 3’ UTR. Library preparation (enzymatic fragmentation, adapter ligation, and barcoding) was performed using the NGSgo-LibrX and NGSgo-IndX kits (GenDx) according to the manufacturer’s instructions. The final denatured library was sequenced using a MiSeq system with a 300-cycle MiSeq Reagent Kit (Illumina). HLA-E genotype determination was performed using NGSengine software (GenDX, version 2.21.0.20156) and the IPD-IMGT/HLA database as a reference (version 3.38.0). Raw sequencing data are available at the European Nucleotide Archive under the accession number ERA18504000.

### Statistical analysis

GraphPad Prism version 7 for Mac OS X (La Jolla, CA) was used for statistical analyses. For comparison between groups, non-parametric Mann-Whitney (MW) test was performed for unpaired data and Wilcoxon test (W) for paired data. χ^2^ analysis was used to detect differences in the frequency of individuals with plasma viral loads below the detection level. Multiple comparisons were performed by one-way ANOVA, two-way ANOVA for unpaired data and Friedman’s test (F) for paired data with *post-hoc* false discovery rate correction (FDR). HLA-E mRNA expression levels measured by RT-PCR were corrected for CD4+ T cell counts when possible. Correlation between two measures was analyzed using the non-parametric Spearman rank test, the corresponding correlation p-value was also calculated. Statistical significance criteria were set at p-value <0.05 and q-value <0.15.

## Results

### Association of HLA-E expression level and allele genotype with HIV disease progression

**Figure 1:**
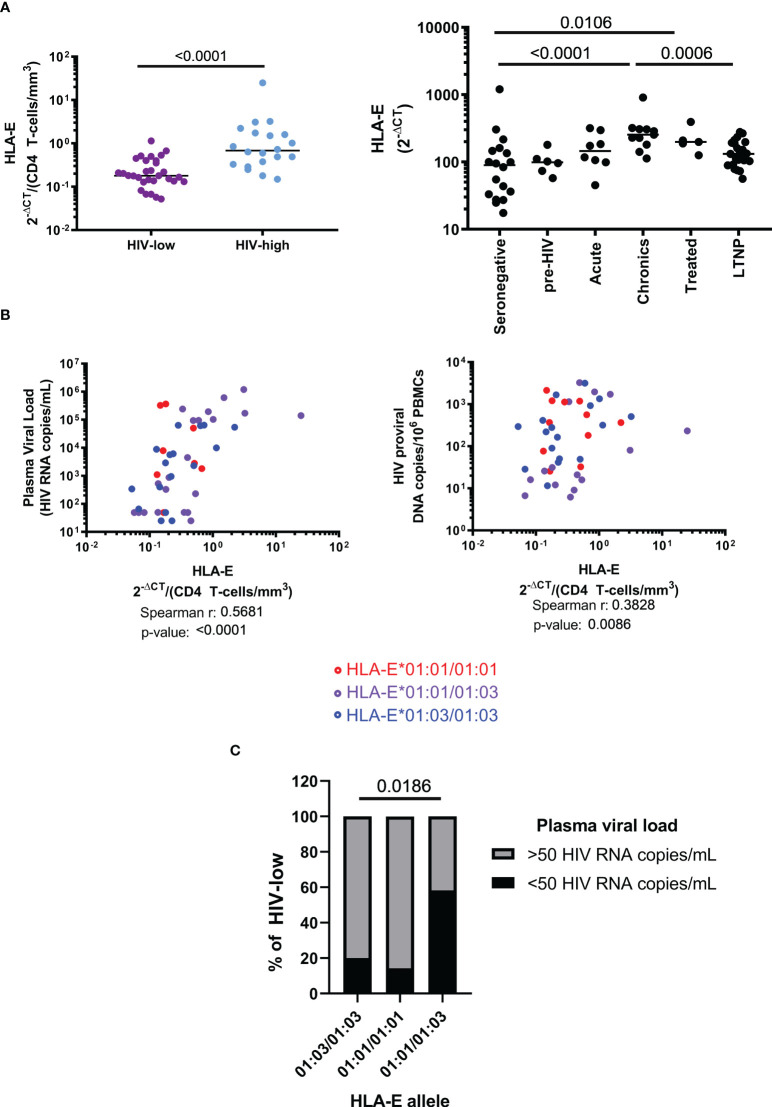
Role of HLA-E allele haplotypes and expression level during HIV infection. **(A)** HLA-E mRNA expression, measured by qPCR and corrected by CD4 count, is reduced in HIV-low individuals (n=31) compared to HIV-high (n=20) individuals (left). HLA-E expression progressively increases during natural course of HIV infection (right). **(B)** HLA-E expression levels correlated positively with plasma viral loads and HIV viral reservoir size (determined as copy number of integrated proviral DNA). HLA-E allelic differences showed no difference in terms of HLA-E expression, plasma viral load and reservoir size. **(C)** Frequency of HLA-E genotypes within the HIV-low group (n=31). Mann-Whitney test was performed to compare differences between groups **(A)**, Spearman’s rank correlation coefficient was applied to evaluate correlations **(B)** and Chi-square was performed to determine the significance of HLA-E haplotype frequencies among HIV natural controllers **(C)**. Results were considered statistically significant when p-value <0.05 while trends are indicated in grey.

In order to detect a potential relationship between HLA-E gene expression and the course of HIV infection, we evaluated the HLA-E mRNA levels in total PBMC from HLA-E genotyped, HIV-seropositive individuals with different levels of viremia and CD4^+^ T cell counts. Significantly different HLA-E mRNA levels were observed between HIV-high and HIV-low groups, even when correcting for CD4^+^ T cell counts (**Figure 1A**, MW p-value <0.0001). This difference was confirmed in unrelated validation cohorts, where a remarkable reduction of HLA-E expression was found in individuals showing control of HIV infection in comparison to untreated HIV infected individuals with high viral loads (MW p-value = 0.0006) (**Figure 1A**). In addition, when compared to HIV seronegative individuals, the levels of HLA-E expression were significantly higher in individuals with chronic HIV infection, receiving cART (MW p-value = 0.0106) or untreated (MW p-value <0.0001). Moreover, we observed not only a direct positive correlation between HLA-E expression and plasma viral load (r = 0.5681, p-value <0.0001), but also between HLA-E expression and proviral HIV copy numbers (r = 0.3828, p-value = 0.0086, **Figure 1B**).

We next clarified whether the single amino acid difference between HLA-E*01:01 and HLA-E*01:03 resulted in differential associations with virological parameters. HLA-E allele haplotype did not impact HLA-E gene expression and did not show any association with HIV plasma viral load when all samples were compared together (data not shown). However, when we compared the impact of the allele polymorphisms between HLA-E*01:01 and *01:03 on plasma viral load only in individuals with lower HIV viral load (HIV-low, n=31), a significantly higher proportion of individuals with undetectable plasma viral load (<50 HIV RNA copies/mL) were HLA-E heterozygous (χ2, p-value = 0.0186, **Figure 1C**). These data suggest that higher levels of HLA-E expression in PBMC are related to uncontrolled chronic HIV infection, while reduced expression is associated with relative control of HIV viremia. In addition, there was a benefit of HLA-E*01:01/01:03 heterozygosity as individuals with low and undetectable viral loads were enriched for a heterozygous genotype.

### Uncontrolled chronic HIV infection is related to an imbalance of the CD56^bright^ NKG2A^+^ and adaptive (CD16^+^CD56^dim^CD57^+^) NKG2C^+^ NK cell subpopulations

In the context of infectious diseases, HLA-E expression has been linked to activation of NK cells with antiviral capacity. Since our analyses indicated that elevated HLA-E expression was related with high plasma viral loads, we examined whether this finding could reflect changes in specific NK cell subsets. For this purpose, we compared the prevalence of NK subsets characterized by the surface expression of NK subset markers CD56, CD16, CD57 and NKG2A and NKG2C in seronegative (SN), HIV-low (HL) and HIV-high (HH) individuals.

**Figure 2:**
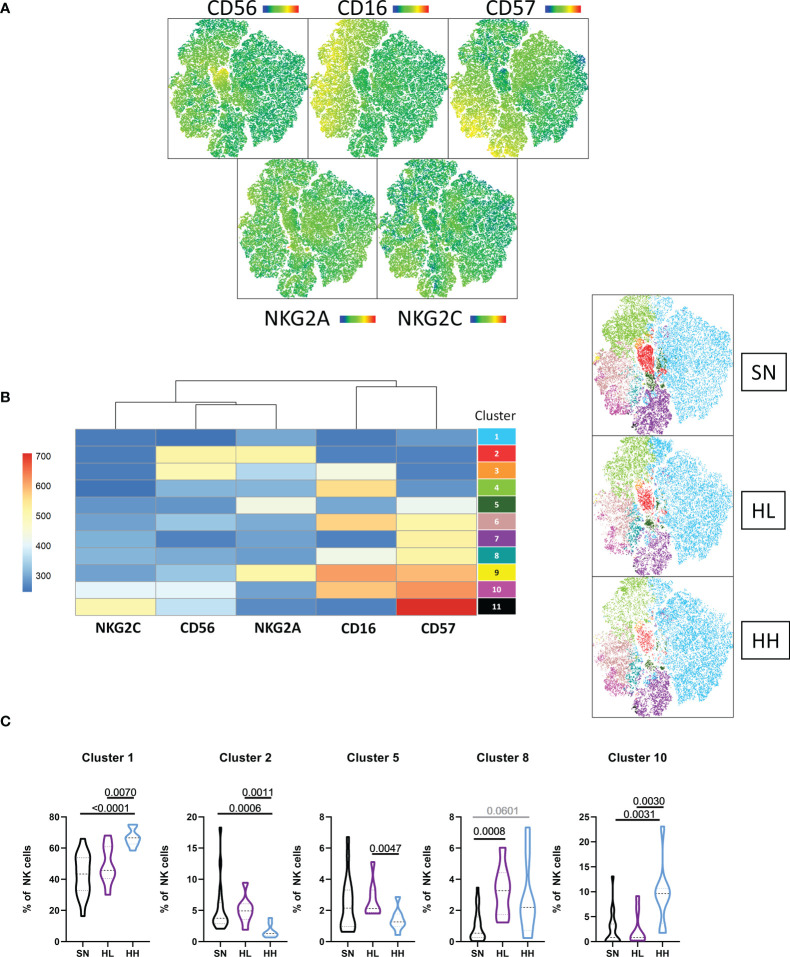
Unsupervised analysis of the NK cell repertoire. **(A)** tSNE dimensional reduction was performed in the CD3 negative fraction of magnetically-sorted NK-cells, based on the expression of CD56, CD16, CD57, NKG2A and NKG2C in a cohort of HIV seronegative individuals (SN, n=18), HIV-low (HL, n=8) and HIV-high (HH, n=8). **(B)** A total of 11 clusters was identified by X-shift and FLOWSOM was applied to determine their specific markers. **(C)** Comparison of clusters differentially represented in the different groups. Comparisons between groups were evaluated by Mann-Whitney test for non-parametric data. Results were considered statistically significant when p-value <0.05 while trends are shown in grey.

Dimensional reduction was conducted after multiparameter flow cytometry based on CD56, CD16, CD57, NKG2A and NKG2C (**Figure 2A**) and 11 clusters were defined. **Figure 2B** represent the relative expression of the surface markers for each cluster. We then compared the abundance of each cluster among groups. Cluster 1 (CD56^-^CD16^-^CD57^-^) was significantly more abundant in HH compared to SN (MW, p <0.0001) and HL (MW, p = 0.0070). By contrast, cluster 2 (CD56^bright^ NKG2A^++^) was significantly reduced in HH compared to SN (MW, p = 0.0006) and HL (MW, p = 0.0011). Cluster 5 (CD56^-^CD16^-^CD57^+^NKG2A^+^NKG2C^-^) was increased in HL compared to HH (MW, p = 0.0047) and cluster 8 (CD56^-^CD16^+^CD57^+^) was increased in HL compared to SN (MW, p = 0.0008). Cluster 10, corresponding to CD16^+^ adaptive NK cells (CD56^+^CD16^++^CD57^+++^NKG2C^+^) were less abundant in SN and HL compared to HH (MW, SN vs HH p-value = 0.0031; HL vs HH, p-value = 0.0030), linking the expansion of these cells to chronic uncontrolled HIV infection (**Figure 2C**). This imbalance in the abundance of CD56^bright^ and adaptive NK cell subsets was also associated with differences in the expression of HLA-E receptors NKG2A and NKG2C, suggesting that HLA-E expression drives this distinct NK differentiation and thus influences their capacity to support control of HIV infection.

**Figure 3:**
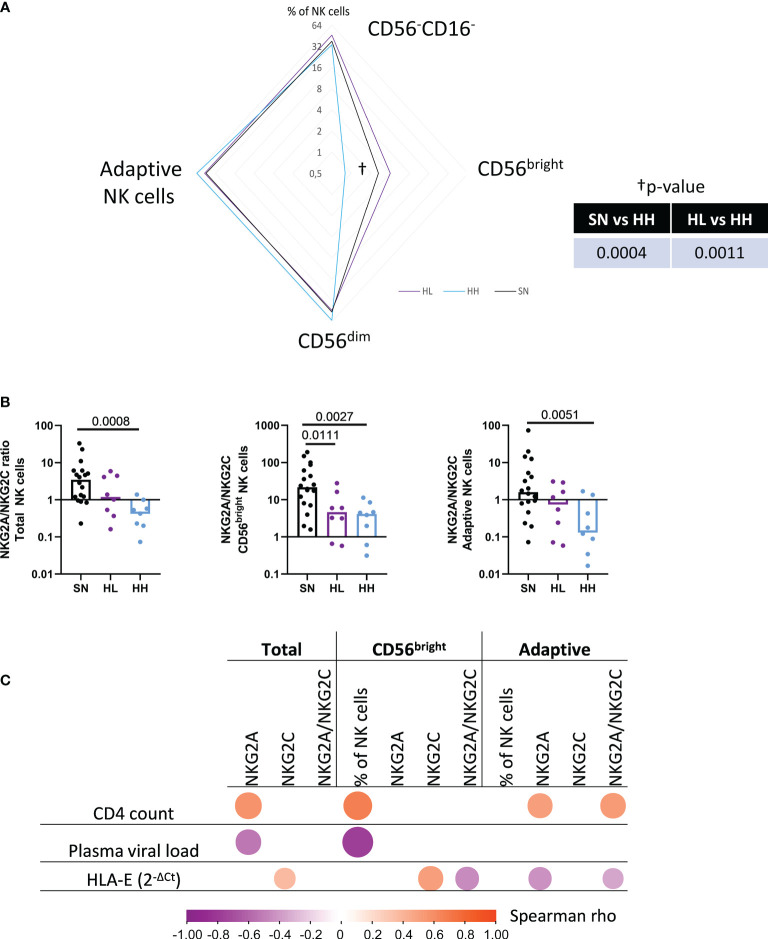
Manual gating analysis of the NK cell fraction. **(A)** Magnetically sorted NK cells from HIV seronegative (SN, n=18), HIV-low (HL, n=8) and HIV-high (HH, n=8) were characterized by manual gating. Four different subpopulations were characterized as CD56^-^CD16^-^, CD56^bright^ (CD56^bright+^CD16^-^), CD56^dim^ (CD56^dim^CD16^+^) and adaptive NK cells (CD56^dim^CD16^+^CD57^+^). Differences between groups (SN, HL, HH) were analyzed by non-parametric Mann-Whitney test and represented in the radar chart. **(B)** NKG2A/NKG2C expression ratio in total NK cell fraction (left), CD56^bright^ (middle) and adaptive NK cells (right) in the different groups (SN-black, HL-purple, HH-blue). **(C)** Correlogram showing Spearman’s rank test correlation of NKG2A and NKG2C expression in total NK cells, CD56^bright^ and adaptive NK cells with clinical parameters (CD4 count, plasma viral load) and HLA-E mRNA expression. Only significant correlations are shown. Results were considered statistically significant when p-value <0.05.

To further explore this hypothesis, the proportions of CD56^-^CD16^-^, CD56^bright^ (CD56^brigth^CD16^-^), CD56^dim^ (CD56^dim^CD16^+^) and adaptive NK (CD56^dim^CD16^+^CD57^+^) populations, together with their expression of the HLA-E receptors NKG2A and NKG2C in SN, HL and HH were determined. The median % of these subpopulations in HL, HH and SN is represented as a radar chart in **Figure 3A**. HH showed a significant loss of CD56^bright^ NK cells compared to HL (MW p-value = 0.0011) and SN (MW p-value = 0.0004). In addition, in total NK cells, the NKG2A/NKG2C ratio was significantly increased in SN compared to HH (MW p-value = 0.0008), highlighting the reduced expression of NKG2A in chronic uncontrolled HIV infection. The subpopulation analysis indicated this difference to stem mainly from CD56^bright^ NK cells (MW p-value = 0.0027) and adaptive NK cells (MW p-value = 0.0051). The shift towards NKG2C expression was less pronounced in HL compared to SN. Still, a significant reduction was observed in NKG2A/NKG2C ratios in CD56^bright^ compared to SN individuals (MW, p-value = 0.0111) (**Figure 3B**). Overall, these data indicate that CD56^bright^ NK cells are reduced in individuals with higher viremia, while adaptive NK cells are more abundant in those samples. NKG2A/NKG2C ratio was related to uncontrolled infections and observed not only in the whole NK cell population, but also in CD56^bright^ and adaptive NK cells subsets.

We next tested for potential relationships between NK cell subsets size, the relative levels of the NKG2A and NKG2C expression in each of these subset as well as the NKG2A/NKG2C ratio and the CD4 count, plasma viral load and HLA-E mRNA levels (**Figure 3C**). CD4 count correlated positively with NKG2A (r=0.61, p-value=0.002) surface expression in total NK cell fraction, NKG2A expression and NKG2A/NKG2C ratio in adaptive NK cells (r=0.51, p-value=0.048 and r=0.57, p-value=0.024, respectively) and the abundance of CD56^bright^ NK cells (r=0.67, p-value=0.006). Plasma viral load correlated negatively with NKG2A expression in total NK cells (r= -0.527, p-value=0.038) as well as with the preservation of CD56^bright^ subpopulation (r= -0.776, p-value=0.001). Of note, HLA-E mRNA correlated positively with NKG2C expression in total NK cell fraction (r=0.367, p-value=0.033) and CD56^bright^ (r=0.508, p-value=0.002) NK cells while it correlated negatively with NKG2A expression in adaptive NK cells (r=-0.427, p-value=0.012). As a consequence, HLA-E expression was correlated with an imbalance in the NKG2A/C ratio in adaptive NK cells (r= -0.344, p-value=0.046), indicating a link between the increased expression of NKG2C, its ligand HLA-E and uncontrolled HIV infection. Furthermore, the results indicate that NK cell subsets and the expression of NKG2X receptors are severely impacted by chronic HIV infection since HIV infected individuals, and individuals with high viral loads (HH individuals) in particular, showed a loss of CD56^bright^ and an increase of the terminally-differentiated adaptive NK cells (characterized here as CD56^dim^CD16^+^CD57^+^). The HLA-E/NKG2X axis also showed severe alterations depending on CD4 count and plasma viral load, suggesting that the impaired antiviral activity of these NK-subsets could be driven by high levels of HLA-E expression during uncontrolled HIV infection.

### NK cells exposed to high levels of HLA-E show an impaired function

**Figure 4:**
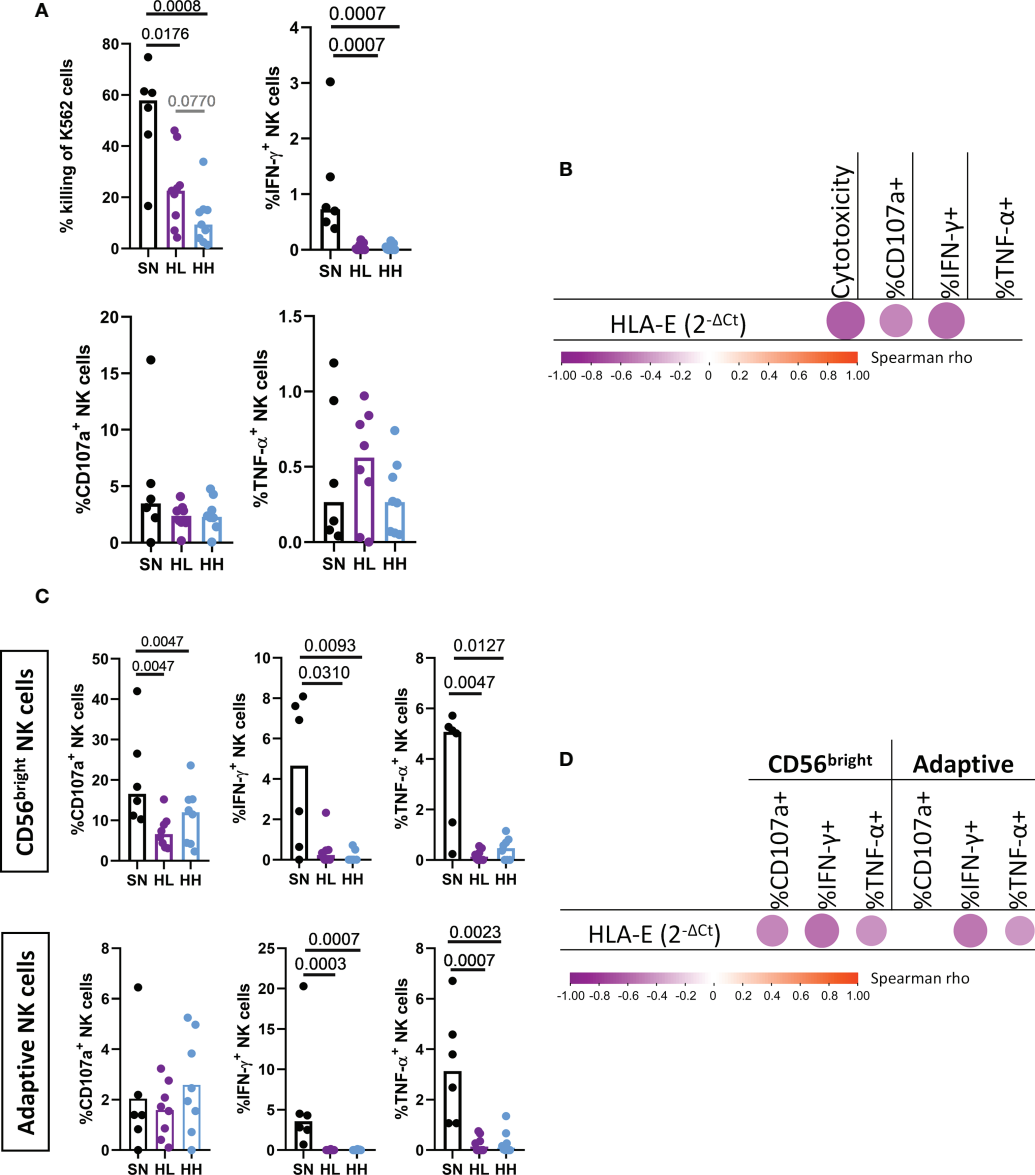
Analysis of NK cell cytotoxic activity, degranulation and cytokine secretion upon HLA-E stimulation. Magnetically sorted NK cells from HIV seronegative (SN, black, n=6), HIV-Low (HL, purple, n=8) and HIV-high (HH, blue, n=8) were cocultured with K562 cell lines (lacking the expression of HLA-E) at an effector:target ratio of 5:1. After four hours, NK functional responses were measured by flow cytometry. **(A)** Total NK cell functions: cytotoxicity against K562 (top left), degranulation measured as % of CD107a^+^ (bottom left), production of IFN-γ (top right) and TNF-α (bottom right). **(B)** Correlogram of HLA-E expression in SN, HL and HH with markers of total NK functionality. **C**) Functional response in the CD56^bright^ (top) and adaptive NK cells (bottom) in terms of degranulation (% of CD107a^+^) (left), production of IFN-γ (middle) and TNF-α (right). **(D)** Correlogram of HLA-E level of expression with CD56^bright^ and adaptive NK cell functions. Comparisons between groups were evaluated by Mann-Whitney test for non-parametric data while correlations were performed by Spearman’s rank correlation test. Results were considered statistically significant when p-value <0.05 while trends are shown in grey.

Given the significant changes observed in NK-cell subset distribution, their NKG2X receptor expression and their correlations with HLA-E expression, we next determined the ability of NK cells isolated from different patient groups to respond to signals through HLA-E/NKG2X. To measure NK response to “missing-self”, we determined cytotoxicity (measured as % of killed K562), degranulation (% of CD107a^+^ NK cells) and cytokine production (IFN-γ^+^ and TNF-α^+^ NK cells) after coculture with HLA-null K562 cells. When testing total NK cells derived from SN, HL and HH, we observed a significant reduction in NK cytotoxic capacity in HIV infected individuals compared with SN, true for both HL (MW p-value = 0.0176) and, especially, for HH (MW p-value = 0.0008, **Figure 4A**). A direct comparison between HL and HH demonstrated stronger NK cell cytotoxic activity in HL, although this did not reach statistical significance (MW, p-value = 0.077). Impaired NK cell function in HIV-infected individuals was also reflected by reduced IFN-γ production in both HL (MW, p-value = 0.0007) and HH (MW, p-value = 0.0007) compared to SN (**Figure 4A**). No differences in degranulation levels nor TNF-α^+^ cells were observed. We tested then if these impairments were associated with the expression of HLA-E by correlating the different NK effector functions with the basal level of HLA-E mRNA expression in the PBMC sample used to isolate the NK cells. Indeed, HLA-E expression levels were associated with a detrimental effect on NK cell function since we observed negative correlations between HLA-E expression and NK cytotoxicity (r= -0.6341, p-value=0.0015), degranulation (%CD107a^+^, r= -0.4647, p-value=0.0293) and IFN-γ production (r= -0.5744, p-value=0.0052, **Figure 4B**).

To gain further insights whether HLA-E expression was associated with dysregulated NK function in specific NK subsets, we repeated the effector function analysis focusing on the two NK subsets showing the most significant alterations in chronic HIV infection, namely CD56^bright^ and adaptive NK cells (**Figure 4C**). The HIV uninfected group (SN) showed indeed superior capacity of CD56^bright^ cells to degranulate (MW, SN vs HL p-value= 0.0047, SN vs HH p-value = 0.0047), secrete IFN-γ (MW, SN vs HL p-value = 0.0310, SN vs HH p-value = 0.0093) or produce TNF-α (MW, SN vs HL p-value = 0.0047, SN vs HH p-value = 0.0127). No significant differences in CD56^bright^ functional responses were observed between HL and HH, although this subset was significantly reduced in HH. Similarly, the adaptive NK subset showed a significantly higher IFN-γ (MW, SN vs HL p-value = 0.0003, SN vs HH p-value =0.0007) and TNF-α production (MW, SN vs HL p-value = 0.0007, SN vs HH p-value = 0.0023) in uninfected individuals compared to chronically HIV infected individuals. Interestingly, degranulation activity was not reduced in this adaptive NK subset which was also not reduced in size in HIV infected individuals. In line with the data obtained in the total NK population, HLA-E mRNA expression in the source PBMC was negatively corelated (**Figure 4D**) with the degranulation capacity of CD56^bright^ (r= -0.4692, p-value=0.0276), IFN-γ and TNF-α production in CD56^bright^ (r=-0.5538, p-value=0.0075; r = -0.4363, p-value=0.0424, respectively) and adaptive NK cells production of IFN-γ and TNF-α (r=-0.5305, p-value=0.0111; r = -0.5598, p-value=0.0067, respectively). Overall, these results indicate that higher levels of HLA-E expression in chronic HIV infection are not only associated with shifts in the NKG2A to NKG2C expression ratios, but also with reductions in cytotoxic, degranulation and cytokine production effector functions in the most affected NK subsets.

### Short-term NK-mediated HIV viral growth inhibition is related to HLA-E expression

**Figure 5:**
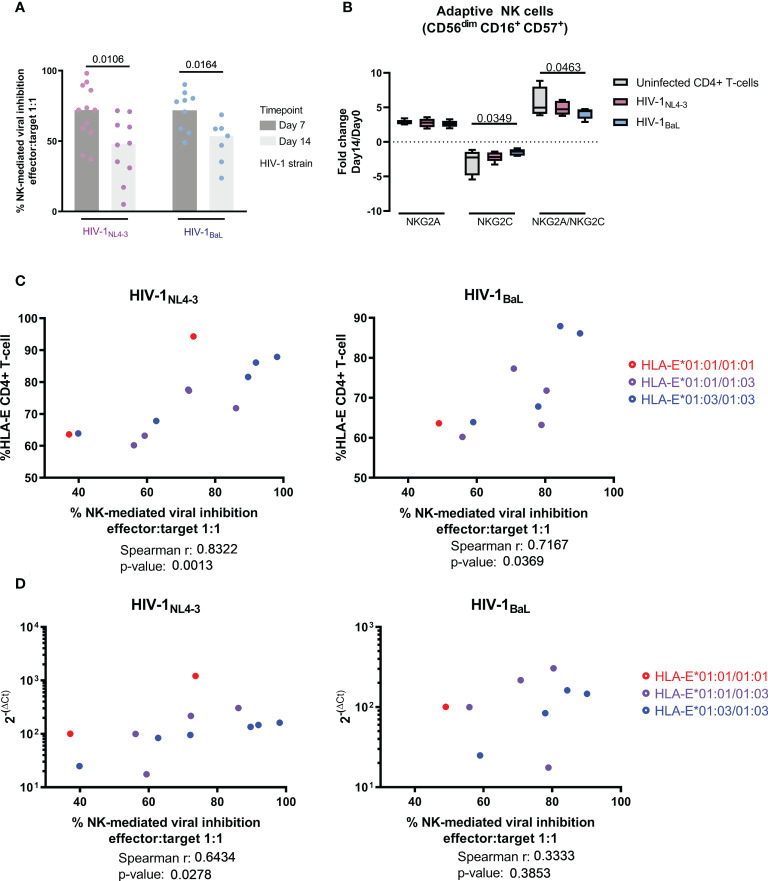
Short term NK-mediated inhibition of viral replication is related to HLA-E expression and diminishes over time. **(A)** Inhibition of viral replication comparing two different laboratory adapted strains (HIV-1_NL4-3_ in pink and HIV-1_BaL_ in blue) and two different timepoints (day 7 in dark grey and day 14 in light grey). **(B)** Long-term NK-CD4^+^ T-cell coculture induced changes on the NK HLA-E receptors favoring the expression of the inhibitor NKG2A. HIV infection resulted in higher levels of NKG2C expression as well as a lower level of NKG2A/NKG2C ratios in adaptive NK cells. **(C)** NK cell control of the viral infection correlated with HLA-E surface expression and basal mRNA levels **(D)**. Color code indicates sample genotype. Univariate analysis was performed using Wilcoxon test for paired data **(A)**, while multiple comparisons were performed by one-way ANOVA **(B)**. Correlation was calculated using Spearman ‘s rank correlation coefficient **(C, D)**. Results were considered statistically significant when p-value <0.05.

In order to discern whether the reduced NK cell effector response was the cause or consequence of uncontrolled HIV infection, we modeled acute HIV infection in an *in vitro* infection model. Using this model, we assessed antiviral activity of NK cells in an *in vitro* viral replication inhibition assay (NK-VIA) by coculturing sorted NK cells from HIV-negative individuals with *in vitro* HIV infected autologous CD4+ T-cells. For the two HIV strains tested, a strong inhibitory capacity of NK cells was observed at day 7 (**Figure 5A**) of culture. However, this activity was significantly reduced by day 14 for HIV-1_NL4-3_ (W p-value = 0.0106) and HIV-1_BaL._ (W p-value = 0.0164). In parallel, the 14-day culture showed marked changes in NK subpopulations, with a loss of CD56^dim^ and a gain of CD56^bright^ and CD56^neg^ subsets (**Figure S2**). The expression of NKG2A was increased in all NK subsets at day 14, while NKG2C was increased in CD56^neg^ and CD56^dim^ (**Figure S3**) but reduced in adaptive NK cells (**Figure 5B**) in line with observations in long-term chronic HIV infection. Furthermore, in adaptive NK cells, coculture with HIV-1_BaL_ infected CD4^+^ T-cells maintained NKG2C expression at higher levels compared to uninfected cultures, reflected by a lower NKG2A/NKG2C ratio (Two-way ANOVA, p-value = 0.0463 **Figure 5B**), suggesting HIV infection might be indirectly driving NKG2C expression on NK cells.

When measuring viral HIV Gag p24 in culture supernatants over time, strong correlations were observed at day 7 between NK-mediated inhibition of viral replication and surface HLA-E expression (**Figure 5C**), for both, HIV-1_NL4-3_ (r= 0. 8322; p-value = 0.0013) and HIV-1_BaL_ (r= 0.7167; p-value = 0.0369). The level of inhibition also correlated with the basal HLA-E mRNA expression in the PBMC used to isolate autologous NK and CD4+ T cells (**Figure 5D**), in the case of HIV-1_NL4-3_ (r= 0.6434; p-value = 0.0278). This was in line with the direct correlation between HLA-E mRNA level in the sample and surface HLA-E expression (**Figure S4**, r= 0,6643; p-value = 0,0219). There was no difference between HLA-E allele genotype and NK-mediate control in our model of acute infection.

Together, these data show that high levels of HLA-E expression mediate at first, an effective viral inhibition by NK cells. However, our data also suggest that long-term exposure to HIV in untreated chronic infection as well as in extended (day 14) co-cultures with infected CD4 T cells, drives NKG2C expression which, in turn, may cause reduced NK functions due to its diminished affinity for HLA-E presented peptides compared to NKG2A (14).

### Effect of HIV-derived peptides on HLAE*01:03 surface stabilization and consequent ability to alter NK cells activation

**Figure 6:**
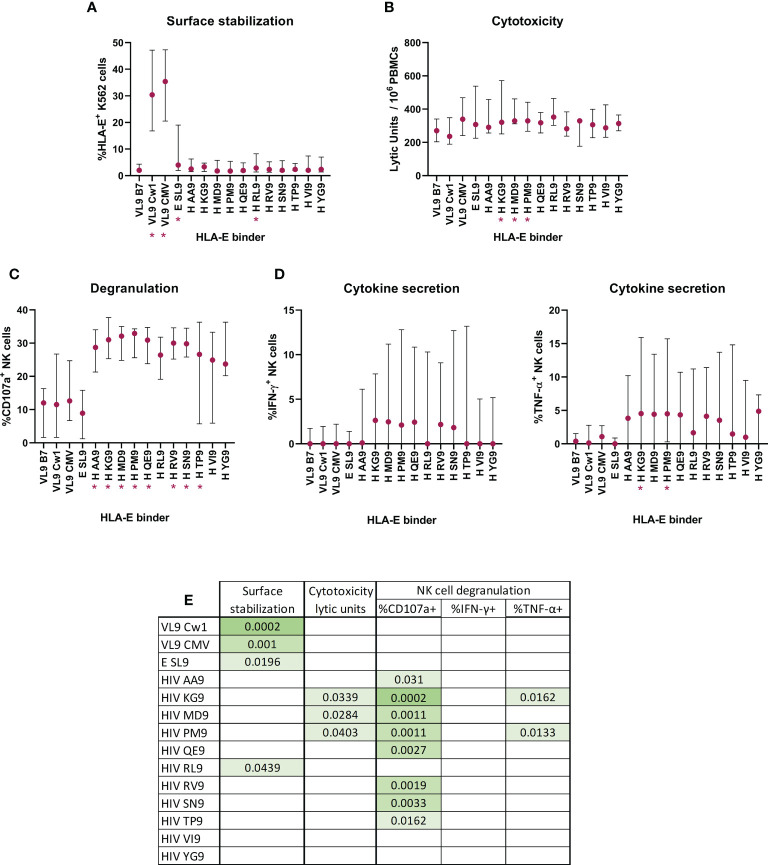
Effect of HIV-1 derived peptides on HLA-E*01:03 surface stabilization and consequent ability to alter NK functionality. HLA-E *01:03 transfected K562 cell line co-expressing the VL9 epitope was pulsed with canonical and non-canonical HLA-E binding peptides. **(A)** Peptide-pulsed cells induced HLA-E surface stabilization, measured by flow cytometry and recorded as %HLA-E+ K562 cells. Peptide-pulsed cells modified the NK cytotoxic capacity **(B)**, degranulation **(C)** and cytokine secretion (**D**, IFN-γ (left) and TNF-α (right)). Multiple comparisons with the control VL9 epitope were performed by Friedman’s test for paired data with *post-hoc* False discovery rate. Significant differences are marked in the graphs with * and **(A-D)** correspond to p-values<0.05. Table **(E)** represents significant p-values when FDR indicated q-values<0.15.

Since HLA-E expression levels were associated with changes in the NKG2A/NKG2C ratio as well as the abundance of the different NK subpopulation, we explored the possibility that a change in the HLA-E peptide repertoire impacts the signaling through the HLA-E/NKG2X axis and drives these alterations. We thus analyzed the role of HIV-derived, HLA-E-binding epitopes in the activation of NK cells from HIV seronegative individuals (n=5), using HLA-E*01:03 transfected K562 cells, co-expressing the canonical peptide VL9-B7 and pulsed with canonical and non-canonical HLA-E binding peptides. We measured HLA-E surface stability as well as HLA-E restricted NK cytotoxicity, degranulation and cytokine production (IFN-γ and TNF-α). The effect of each peptide compared to the canonical, endogenously expressed, VL9-B7 epitope was determined and compared between peptides (**Figure 6**).

The presence of the described canonical HLA-E binding epitopes increased the surface expression when compared with VL9-B7 endogenous expression (Friedman test, VL9-Cw1 p-value = 0.0002, CMV p-value = 0.001). The same was seen for the non-canonical EBV-derived SL9 peptide (Friedman test, E SL9, p-value = 0.0196). Interestingly, only one of the 11 HIV-derived candidate epitopes (H RL9) increased the surface expression (Friedman test, p-value = 0.0439, **Figure 6A**).

Given the different potency of described HLA-E presented peptides to stabilize surface expression, we assessed whether these differences would translate to more or less effective sensitization of target cells to NK cell recognition. Indeed, HLA-E*01:03 expressing K562 target cells pulsed with three HIV-derived peptides increased the cytotoxic activity of NK cells significantly (Friedman test, KG9 p-value = 0.0339, MD9 p-value = 0.0284 and PM9 p-value = 0.0403, **Figure 6B**). These three and all but two exogenously added HIV-derived epitopes triggered significant increases in NK degranulation (Friedman test, AA9 p-value = 0.0310, KG9 p-value = 0.0002, MD9 p-value = 0.0011, PM9 p-value = 0.0011, QE9 p-value = 0.0027, RV9 p-value = 0.0019, SN9 p-value = 0.0033, TP9 p-value = 0.0162, **Figure 6C**). Finally, two of the three HIV-derived peptides that induced cytotoxicity also were able to increase the number of TNF-α^+^ NK cells (Friedman test, KG9 p-value = 0.0162 and PM9 p-value = 0.0133, **Figure 6D**).

Together, eight of the eleven tested HIV peptides induced increases in at least one of the tested NK functions when presented by HLA-E*01:03. Remarkably, two of the HIV peptides (KG9 and PM9) presented by HLA-E*01:03 stimulated NK cells and increased three of the four cellular functions measured (cytotoxicity, degranulation and TNF-α production). These data indicate that HLA-E*01:03 might be sensitive to changes in the HLA-E repertoire and undergo changes that can be recognized by NKG2X receptors on NK cells.

### Structural model of the antigen presentation of HIV-derived peptides through HLA-E to NK cells

**Figure 7:**
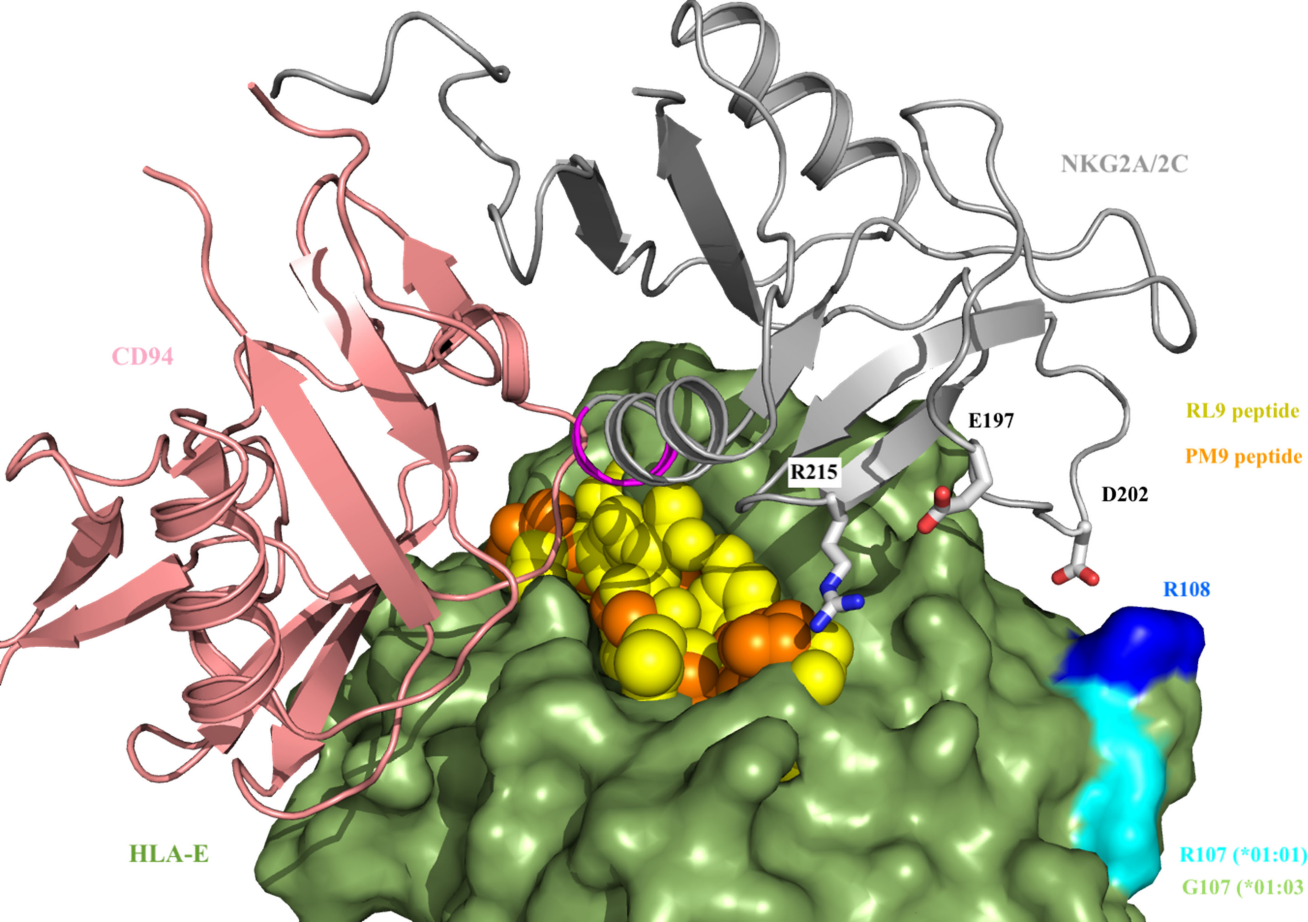
CD94-NKG2(A/C) interaction with HLA-E-VL9/PM9 peptide. VL9 (in yellow) and PM9 (in orange) peptides protrude from HLA-E (in green) allowing for an additional interaction with Arg215 from NKG2A/2C (in grey). Residue 197 (Glu residue in NKG2A and Lys residue in NKG2C, respectively) is close to Arg 215. The ASILP and SIISP sequence motifs (in magenta) presented by NKG2A and NKG2C, respectively, are in close proximity to position P5 of VL9 and PM9/RL9 peptide. HLA-E (in green) differs in Arg/Gly 108 (in cyan), which interacts with NKG2A/2C Asp 201 through Arg 108 (in blue).

In order to further understand the interplay between HLA-E alleles (*01:01 or *01:03), NKG2X receptors and HIV-derived peptides that triggered NK responses, a structural model was constructed. The structural model of HLA-E-CD94-NKG2X (**Figure 7**) illustrates that the presentation of HIV-derived PM9 and RL9 peptides differs in the binding mode compared to canonical VL9 peptides. In particular, the first residue (P1) in both, the PM9 and RL9 epitopes is exposed to NKG2A, providing evidence for a direct interaction between the presented epitope and NKG2X position Arg215, which localizes close to position 197 in NKG2A and NKG2C, which is occupied by a Glu and Lys residue, respectively. Additionally, position P5 in the presented epitope points towards the CD94-NKG2A/C interface and thus, differences in NKG2X affinity for the HLA-E/epitope complex may be determined by the ASILP motif in NKG2C/SIISP in NKG2A that change the CD94 interface, similarly to what happens when the canonical VL9 peptide is presented (49). Interestingly, HLA-E*01:01 and *01:03 differ only in residue Arg/Gly 107, which is outside the epitope binding pocket. It is however located next to another charged residue (Arg108) which is responsible for the interaction with NK cells receptors NKG2A and NKG2C through Asp202 (**Figure 7**). Hence, the NKG2A and NKG2C molecules can be expected to be very sensitive to minor changes in HLA-E allotypes affecting this position (Arg108) (**Figure 7**).

Thus, our structural model identifies interactions between non-canonical HIV-1-derived HLA-E presented peptides and NKG2X receptors in an allele-dependent manner, providing the potential explanation for differential NK-cell recognition of infected cells through the HLA-E/NKG2X axis. In this model, Position 5 in the presented epitope may further determine the affinity to the activator (NKG2C) or inhibitory (NKG2A) receptor.

## Discussion

NK cells play a crucial role in antiviral immunity, a role evidenced by the increased susceptibility to viral infections when NK cells are impaired or depleted (38, 40, 50). Aside from surface molecules such as Fc-receptors that are involved in antibody-mediated effector functions, NK exert a large portion of their activity by detecting changes in HLA surface expression or in the HLA-presented epitope repertoire, caused by viral infections. The importance of these defense mechanisms is supported by the observation that viruses causing chronic infections, like CMV, EBV and HIV, have developed several mechanisms to escape such NK responses, including the reduction of HLA expression on infected cells, expressing epitopes mimicking human HLA canonical epitopes or influencing NK subsets and their immune-receptor repertoire (25, 26). Of these NK receptors, the interaction between NKG2X and HLA-E on the surface of target cells is especially interesting, given the conserved nature of the HLA-E locus compared to classical HLA class I loci (HLA-A, -B, or –C) and the variable expression of different NKG2X throughout the NK differentiation process. The conserved nature of HLA-E has also the potential to render NK-mediated recognition and elimination of virally infected cells through HLA-E an antiviral mechanism that extends across all the human population, not restricted to individuals expressing a specific classical HLA class I genotype. For these reasons, in the present study we aimed to determine the relevance of NK cell activation through the HLA-E/NKG2X axis in the natural control of HIV infection.

Previous studies reported a link between HLA-E expression and HIV infection and showed that infected CD4^+^ T-cells had an increased surface HLA-E expression compared to their uninfected counterparts (51). In our initial study population, we observed a progressive increase of HLA-E mRNA expression along the HIV disease course and documented a strong positive correlation between increased HLA-E expression and plasma viral loads and proviral DNA in cART-naïve individuals with chronic HIV infection. While the precise mechanisms of such increased expression remain to be defined, it is possible that different soluble factors, such as IL-27, could be potential drivers. In fact, it has been demonstrated that IL-27 is i) capable of upregulating surface expression of HLA-E (52), ii) significantly increased in individuals with high plasma viremia (35) and iii) associated with ineffective NK-cell responses (36). Such an effect may not be limited to drive HLA-E expression on infected T cells, but may also extend to other cell types susceptible to HIV infection, such as macrophages and other cells, chronically exposing NK to sustained elevated levels of HLA-E.

Importantly, in our study we also controlled for HLA-E allelic differences in terms of HIV disease progression and NK cells activation. This aspect has been largely neglected in the past, probably due to the fact that the two worldwide distributed alleles, HLA-E*01:01 and *01:03, only differ in one amino acid. However, it is well known that such small differences can cause functional differences and, for instance in the case of HLA-B*58, have been associated with opposite risk for HIV disease progression (37). Indeed, in our cohort, HLA-E heterozygosity (HLA-E*01:01/01:03) was significantly more common in individuals with naturally controlled HIV infection and with undetectable viral loads. Such an advantage of heterozygous HLA genetics has been described in the past for classical HLA class I alleles and for rare allele variants (34), but not for the less polymorphic HLA-E locus. This observation also suggests that the functional differences between the two alleles may be pronounced enough to provide heterozygous individuals an advantage to control HIV more effectively than HLA-E homozygous individuals. The lack of K562 cells expressing HLA-E*01:01 only, prevented us from further clarifying the inherent ability of each allele to be stabilized on cell surface by binding epitopes.

A past study by Natterman et al. also demonstrated that HIV infection led to reduced NK cell activity (51). To understand whether and how higher HLA-E levels were related to reduced HIV control, we investigated the relationship between the potential to activate NK cells through the HLA-E/NKG2X axis and controlled and uncontrolled HIV infection. We observed that the HLA-E/NKG2X axis was profoundly impacted by chronic HIV infection, especially among individuals with high viral loads. In particular, NK cells from HIV infected individuals showed a severely impaired activation capacity through HLA-E “missing-self” mechanism as they were less activated when exposed to target cells lacking HLA-E. We also found that HIV infected individuals, and especially HIV-high, showed a switch of the HLA-E receptors NKG2A/NKG2C ratio, in line with previous studies (27). Thus, our data suggest that increased expression of NKG2C, together with the chronic nature of HIV infection, causes constant activation of NK cells, ultimately leading to an impaired state. HLA-E is thought to play into this mechanisms by its relative lower affinity of NKG2C compared to NKG2A, causing loss of NK regulation (14).

This interpretation is also in line with the findings of Merino et al (5), who showed that chronic *in vitro* stimulation of NK cells through the HLA-E/NKG2X axis, using plate-bound agonistic antibodies, promoted the expansion of adaptive NK cells and induced the expression NKG2C and CD57. These cells also present an epigenetic reprogramming that causes an exhausted phenotype and profound NK dysfunction (5), which may be difficult to be restored (53). In other infections like HCMV, NK subset shifts are equally associated with an epigenetic imprint that modulates NK cells function and drives the expansion of mature, memory-like, NKG2C^+^CD57^+^ NK cells (26, 54). In our case, we observed that uncontrolled HIV infection in the absence of ART was associated with a similar increase in HLA-E mRNA expression and loss of the highly active CD56^bright^ NK subset, accompanied by an expansion of terminally differentiated, adaptive NK cells. To validate these observations in HIV infection, we extended our analyses to HIV-uninfected individuals and found common patterns in NK cells subsets between HIV seronegative and HIV-low that differed from those in HIV-high individuals. This included the maintenance of NKG2A expressing CD56^bright^ NK cells and a reduction in the frequency of the terminally differentiated NKG2C^+^ adaptive NK cells compared to highly viremic individuals. Notably, we also observed a cluster of CD56^dim^CD57^bright^NKG2C^+^ which was increased in HIV patients compared to uninfected samples, suggestive of a progressive increase of NKG2C^+^ adaptive NK cells and a loss of CD56^bright^ NK cells in progressive HIV disease. To validate these findings, we measured the expression of NKG2A and NKG2C in four different NK cells subpopulations defined as CD56^-^CD16^-^, CD56^bright^, CD56^dim^ and adaptive NK cells (CD56^dim^CD16^+^CD57^+^). We confirmed that HIV-high showed a profound loss of CD56^bright^ NK cells and identified a significant reversion of the NKG2A/NKG2C balance in total NK cells from HIV infected individuals’ cells, which was mainly driven by the CD56^bright^ and adaptive subsets. While some preceding studies reported NKG2C^+^NKG2A^-^ NK cells as a marker of lower viral set point (55) and an increased killing of HIV infected CD4^+^ T-cells by autologous NK cells when blocking NKG2A (51), both studies were based on acute HIV infection *ex vivo* or *in vitro*, respectively. Still, both studies established a link between the course of HIV disease and HLA-E/NKG2X regulation of NK cell activity.

Our results are in line with the observed reversion of the NKG2A/NKG2C ratio in NK cells from HIV infected individuals with HCMV co-infection (27). HCMV is a highly disseminated virus within the human population, with an estimated worldwide seroprevalence of 83% (95%UI: 78-88) (56). Although we could not determine the CMV status for all participants in the present study, its high prevalence suggests that the vast majority of these samples were HCMV infected as well and some of the alterations on NK cell phenotype and function could also be related to latent CMV infection. We observed an impaired NK cell response upon stimulation through missing self paradigm along with this consistent switch from NKG2C to NKG2A expression. Further studies assessing NK cell exhaustion in a defined co-infection cohort will be needed to conclusively determine the functional consequences of the reverted receptor ratio.

In our ex-vivo model of NK mediated inhibition of HIV replication, we observed a positive correlation between NK-mediated antiviral activity and HLA-E expression within the first seven days of culture. After fourteen days of culture however, NK mediated viral control was significantly reduced and the correlation with HLA-E expression was lost. We observed a shift towards NKG2C expression in HIV-1_BaL_ infected cultures concomitant with a reduced NK killing activity in longer-term cultures. These results are in line with previous *in vitro* HIV studies, were the continuous stimulation through the HLA-E/NKG2X axis reduced NK functionality (5). Importantly, the *in vitro* data directly support our ex-vivo data and a scenario where elevated HLA-E expression in response to HIV infection will drive a shift in the NKG2A/NKG2C balance on NK cells, which eventually will hamper their ability to exert effective antiviral activity.

As HLA-E surface expression levels as well as signaling *via* NGK2X receptors can be influenced by the epitope-repertoire presented by HLA-E, we tested whether HIV-derived epitopes presented by HLA-E could affect NK cells activation, potentially by displacing the canonical VL9 epitope and thus leading to NK-mediated cell killing by the “missing-self” mechanism. We first used single HLA-E transfected K562 cells, pulsed with canonical and non-canonical HIV-1-derived peptides and competing with an endogenously expressed VL9-B7 epitope. Although only one HIV-derived peptide increased HLA-E surface levels, we observed profound differences in the activation of NK cells derived from HIV seronegative individuals in response to peptide-pulsed target cells, suggesting that low levels of HLA-E viral antigen presentation could trigger NK cell activation as have been shown for classical HLA class I molecules (57). These analyses could not be repeated in NK cells from HIV infected individuals due to the high cell demand for such assays. Furthermore, our results did not discriminate whether this activation occurs through the abrogation of NKG2A engagement and the consequent missing-self signaling or through NKG2C signaling or a combination of both mechanisms. Further studies including specific receptor blocking may help to determine the exact mechanism of NK cells sensing of HIV infected cells through the HLA-E/NKG2X axis.

Finally, we tested structural models to structurally support the functional impact of HLA-E repertoire, NKG2X receptor switch and HLA-E allele polymorphism on NK cells mediated control of HIV infection. Our analyses indicate that the differences between NKG2A and NKG2C influences the interaction with HLA-E alleles and the bound epitopes in different ways: (1) through the ASILP/SIISP motif on NGK2X that interacts with position P5 of the bound epitope and which could directly determine the affinity to the activator (NKG2C) or inhibitory (NKG2A) receptor to the HLA-E/epitope complex; (2) through E/K 197 residues that map close to presented epitopes, such as PM9 and RL9, and (3) through Asp202 that interacts with Arg 108, located in close proximity to Arg107 and representing the only change between HLA-E*01:01 and *01:03. Some of these structural particularities may support the benefits of HLA-E heterozygosity in HIV control as heterozygous might have a broader and more differentiated ability to present more/different epitopes and thus trigger differential NK responses. The expanded structural insights in the present study may also explain the reported 6-fold lower affinity for the HLA-E-peptide complex to NKG2C and contribute to the observed switch from NKG2A to NKG2C expression, with its downstream functional consequences on NK-mediated antiviral activity.

Overall, our data are in line with a mechanism where chronic HIV infection with high HLA-E expression levels, which is especially pronounced in individuals with uncontrolled HIV infections, continuously stimulates NK cells through the NKG2X receptors, driving a change in NK subpopulations ratios from CD56^bright^ NK cells towards a functionally less active profile. These CD56^bright^ NK cells are traditionally considered as regulatory and their role in HIV control could go beyond the direct killing of infected cells (58). These changes appear to go hand in hand with a profound disruption of the regulation of the “missing-self” NK activation pathway through the HLA-E/NKG2X axis, which can be further influenced by changes in the HLA-E presented epitope repertoire due to HIV infection. Indeed, HL maintained higher levels of cytotoxicity and TNF-α production upon HLA-E stimulation compared to HH although there were not significant differences in NK cell degranulation. This could indicate a mechanism of cell death induction that might differ from the release of cytolytic granules. Our results are further supported by previous studies that have suggested different effects of HIV viremia on NK cell number and function during either acute or chronic stages of the infection in a HLA-E dependent manner (56). Our data are also in line with studies showing NK cells from HIV infected individuals to be in an unresponsiveness state and express transcriptomic signatures indicative of higher exhaustion markers (59), loss of surface NK cytotoxicity receptors expression (60) and a consequent reduction in cytolytic capacity, despite showing elevated levels of activation markers (61). This dysfunctionality has been proposed to contribute to the loss of HIV control, and which may not be readily restored upon ART treatment (62). These findings are especially relevant for potential therapeutic applications targeting NK cells such as enhancing antibody dependent cell cytotoxicity (ADCC)^67^, enhancement of NK activity using TLR ligands^68,69^ or the infusion of engineered NK cells expressing CAR receptors (35). Indeed, there are some reports indicating that NK cells function can be rescued with the addition of IL-15 (63), at least *in vitro*, and also that the exhausted transcriptomic signature observed in uncontrolled HIV infection could be partly restored after vaccination (60). The functional data in our present study and the novel structural insights into how HLA-E allele polymorphism can impact NKG2X receptor recognition may thus allow to therapeutically improve the antiviral capacity of NK cells and to guide future therapeutic use of these cells in HIV cure strategies.

## Data availability statement

The data presented in the study are deposited in the European Nucleotide Archive (ENA) repository, accession number ERA18504000.

## Ethics statement

The studies involving human participants were reviewed and approved by Comitè Ètic d’Investigació Clínica of Hospital Germans Trias i Pujol (CEIC EO-12-042). The patients/participants provided their written informed consent to participate in this study.

## Author contributions

Conceptualization: L-RM, MR-R, CB, AO. Methodology: LR-M, CD-C, MO, MR-U. Investigation: LR-M, CD-C, MR-U. Visualization: LR-M, CB, AO. Supervision: CB, AO. Resources: BM, CB, DH-O'C. Writing – original draft: LR-M. Writing – review & editing: CD-C, MO, MR-U, MR-R, JS, BM, DH-O'C, AO, CB. All authors contributed to the article and approved the submitted version.

## Funding

The present study was supported by the USA National Institutes of Health (NIH) - National Institute of Allergy and Infectious Diseases - P01 Research Program Project Grant 1P01AI131568-01 (CB) and funding from the European Union’s Horizon 2020 research and innovation program under grant European AIDS Vaccine Initiative 2020 (EAVI2020) #GA681137 (CB). The work was also partly supported by grant PI17/01465 (AO) from the Instituto de Salud Carlos III, co-financed by the Fondo Europeo de Desarrollo Regional (FEDER) “Una manera de hacer Europa”. CB is a senior ICREA research professor.

## Acknowledgments

We would like to thank the team of Dr. J. Miguel López-Botet (Departament de Ciències Experimentals i de la Salut, UPF/IMIM, Barcelona) for their advice starting this project.

## Conflict of interest

The authors declare that the research was conducted in the absence of any commercial or financial relationships that could be construed as a potential conflict of interest.

## Publisher’s note

All claims expressed in this article are solely those of the authors and do not necessarily represent those of their affiliated organizations, or those of the publisher, the editors and the reviewers. Any product that may be evaluated in this article, or claim that may be made by its manufacturer, is not guaranteed or endorsed by the publisher.
