# Supplementary figures and images for: Disruption of the HLA-E/NKG2X axis is associated with uncontrolled HIV infections
Source: Front Immunol. 2022 Nov 18;13:1027855. doi: 10.3389/fimmu.2022.1027855 (PMC9716355; doi:10.3389/fimmu.2022.1027855)

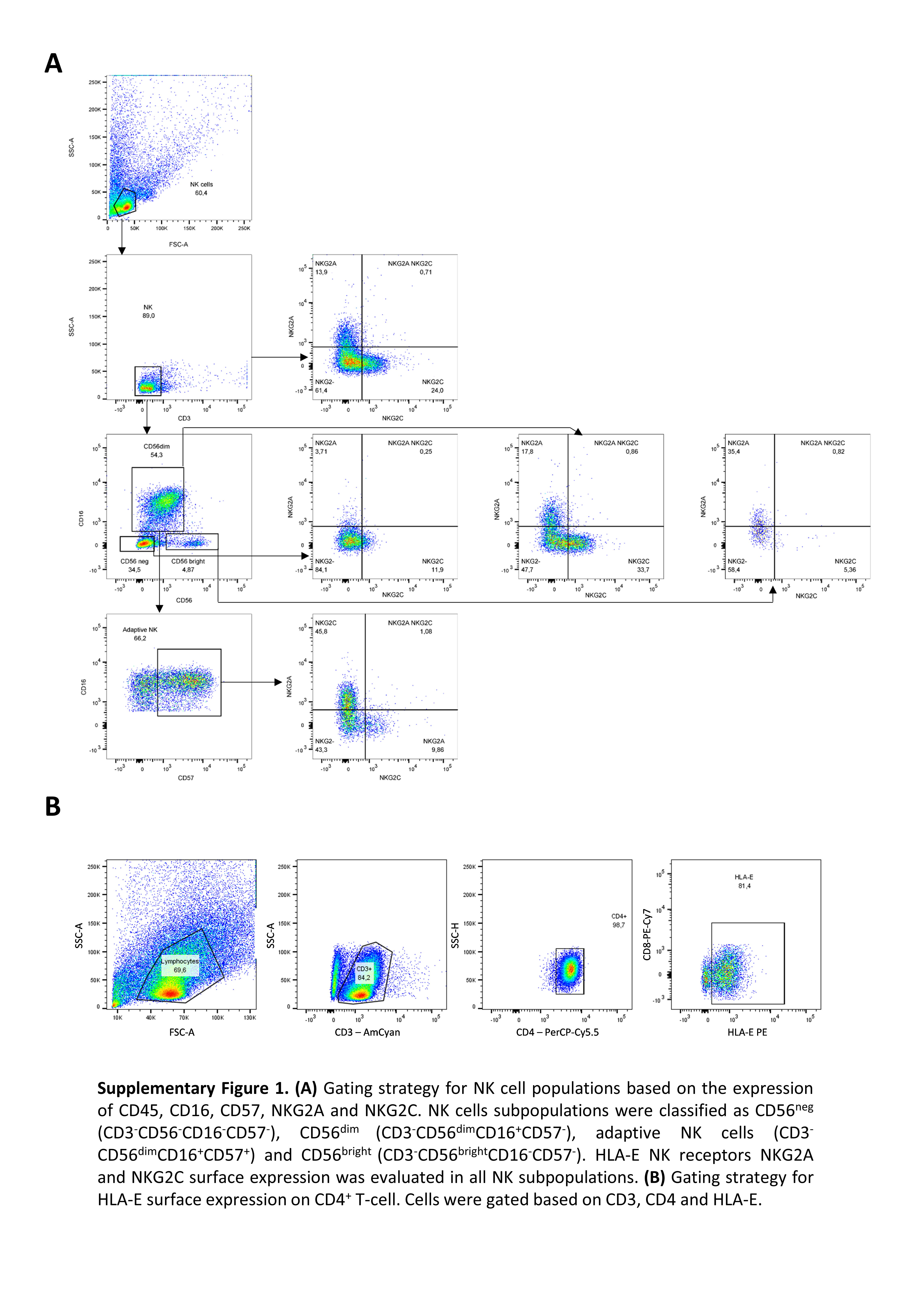

Supplement: Supplementary file 1 [file Image_1.tif]

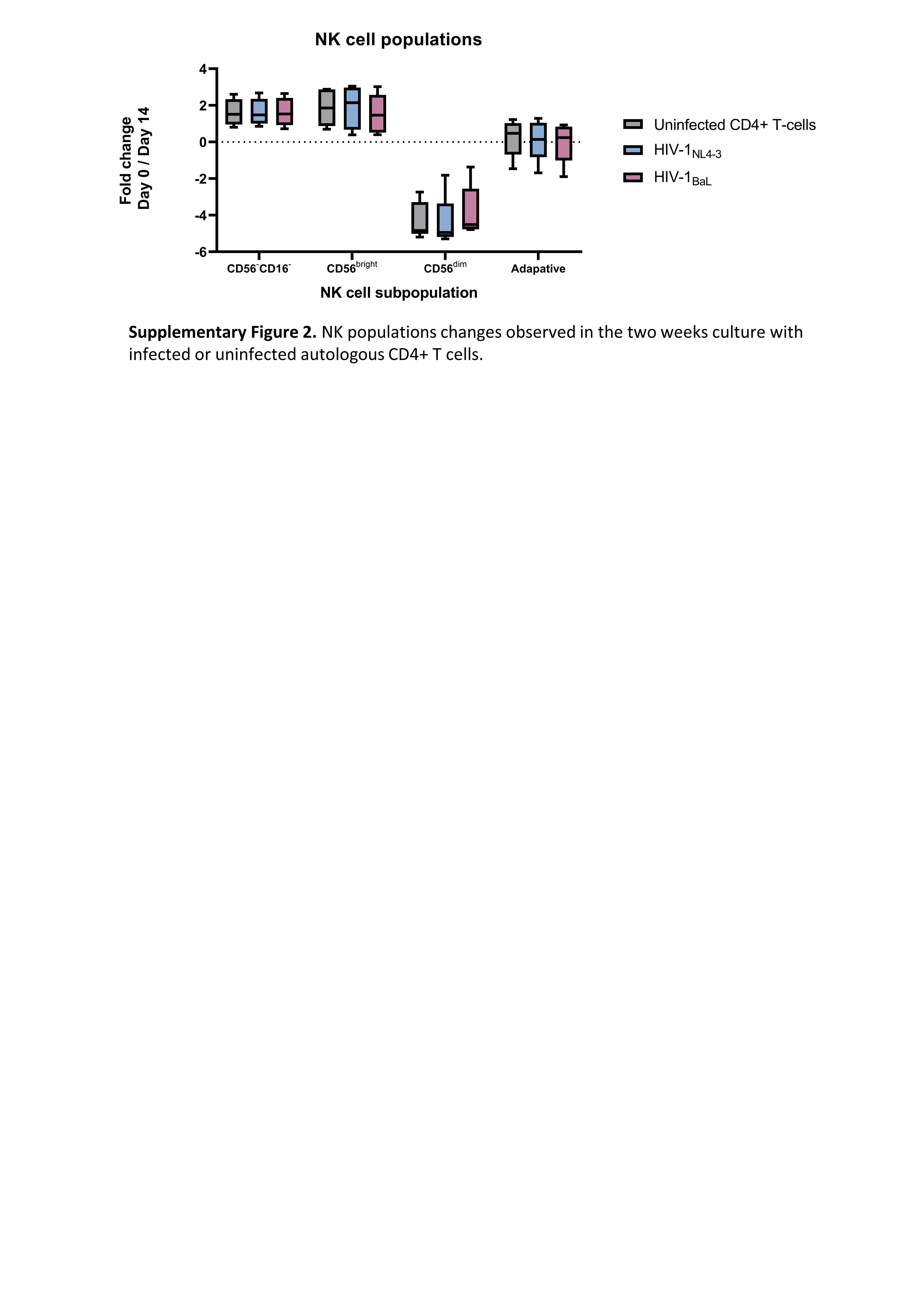

Supplement: Supplementary file 2 [file Image_2.tif]

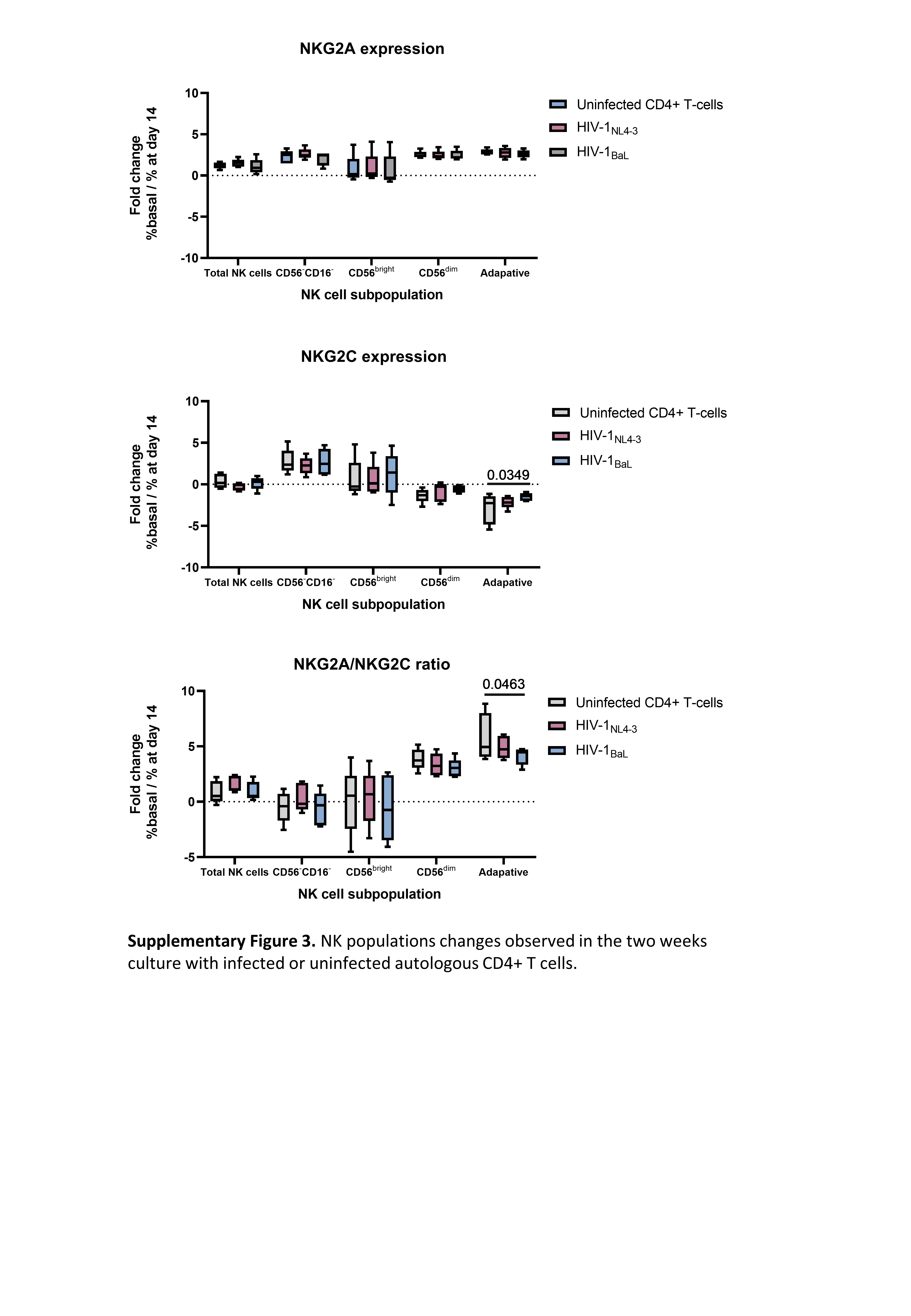

Supplement: Supplementary file 3 [file Image_3.tif]

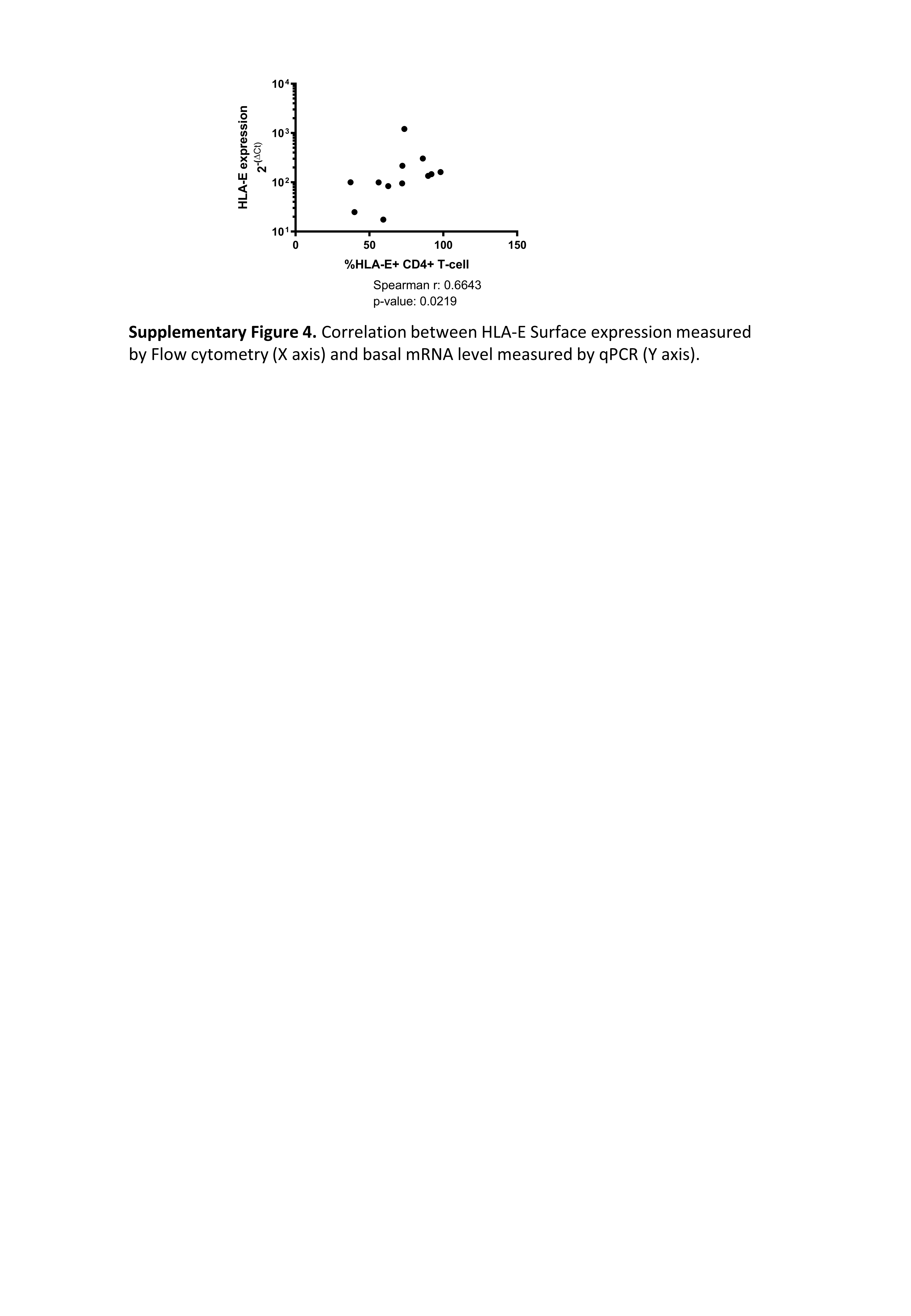

Supplement: Supplementary file 4 [file Image_4.tif]
