# Supplementary material for: Disruption of the HLA-E/NKG2X axis is associated with uncontrolled HIV infections
Source: Front Immunol. 2022 Nov 18;13:1027855. doi: 10.3389/fimmu.2022.1027855 (PMC9716355; doi:10.3389/fimmu.2022.1027855)
Supplement: Supplementary file 5 [file Table_1.docx]

**Supplementary Table 1. HIV-high and HIV-low samples included in this study. Abbreviatures: M: Male, F: Female, N/D: not determined**
